# Automated quantification of Haller’s layer in choroid using swept-source optical coherence tomography

**DOI:** 10.1371/journal.pone.0193324

**Published:** 2018-03-07

**Authors:** Sushmitha Rao Uppugunduri, Mohammed Abdul Rasheed, Ashutosh Richhariya, Soumya Jana, Jay Chhablani, Kiran Kumar Vupparaboina

**Affiliations:** 1 Dept. of Electrical & Electronics Engineering, Birla Institute of Technology and Science, Pilani - Hyderabad, Telangana, India; 2 School of Medical Sciences, University of Hyderabad, Hyderabad, Telangana, India; 3 Engineering Group, Srujana Centre for Innovation, L. V. Prasad Eye Institute, Hyderabad, Telangana, India; 4 Dept. of Electrical Engineering, Indian Institute of Technology Hyderabad, Telangana, India; 5 Smt. Kanuri Santhamma Retina Vitreous Centre, L. V. Prasad Eye Institute, Hyderabad, Telangana, India; Universita degli Studi di Firenze, ITALY

## Abstract

**Objective:**

To develop an algorithm for automated quantification of Haller’s layer in choroid using swept-source optical coherence tomography (OCT).

**Background:**

So far, to understand the association of various diseases with structural changes of choroid, only gross indicators such as thickness, volume and vascularity index have been examined. However, certain diseases affect specific sublayers of the choroid. Accordingly, a need for targeted quantitation arises. In particular, there is significant interest in understanding Haller’s layer, a choroidal sublayer comprising relatively large blood vessels. Unfortunately, its intricate vasculature makes, manual quantitation difficult, tedious, and error-prone. To surmount this difficulty, it is imperative to develop an algorithmic method.

**Methodology:**

The primary contribution of this work consists in developing an approach for detecting the boundary between Haller’s and Sattler’s layers, the latter comprising medium-sized vessels. The proposed algorithm estimates vessel cross-sections using exponentiation-based binarization, and labels a vessel large if its cross-section exceeds certain statistically determined threshold. Finally, the desired boundary is obtained as a smooth curve joining the innermost points of such large vessels. On 50 OCT B-scans (of 50 healthy eyes), our algorithm was validated both qualitatively and quantitatively, by comparing with intra-observer variability. Extensive statistical analysis was performed using metrics including Dice coefficient (DC), correlation coefficient (CC) and absolute difference (AD).

**Results:**

The proposed algorithm achieved a mean DC of 89.48% (SD:5.03%) in close agreement with the intra-observer repeatability of 89.12% (SD:5.68%). Corresponding mean AD and mean CC were of 17.54 *μ*m (SD:16.45*μ*m) and 98.10% (SD:1.60%) which too approximate the respective intra-observer repeatability values 19.19 μm (SD:17.69 μm) and 98.58% (SD:1.12%).

**Conclusion:**

High correlation between algorithmic and manual delineations indicates suitability of our algorithm for clinically analyzing choroid in greater finer details, especially, in diseased eyes.

## Introduction

The choroid consists of three sub-structures: choriocapillaris, Sattler’s layer and Haller’s layer [1]. Choriocapillaris, the innermost structure adjoining retinal outer boundary defined by Bruch’s membrane, comprise the tiniest choroidal vessels. In contrast, Haller’s layer, comprising the largest vessels is the outermost and adjoins the choroid-sclera interface (CSI). Sandwiched in between lies Sattler’s layer comprising medium-sized vessels. Structural changes in choroid potentially indicate various diseased conditions including choroid neovascularization (CNV), high myopia, chorioretinal inflammatory diseases, tumors, central serous chorioretinopathy (CSC), age-related macular degeneration (AMD) and diabetic retinopathy (DR) [2, 3, 4, 5, 6, 7, 8, 9]. Against this backdrop, understanding the structural changes in choroid and their relevance to various diseases as well as disease management has been of long-standing interest to clinicians. Indeed, ophthalmologists now envision diving deeper and examining at sub-structural levels [10].

**Fig 1:**
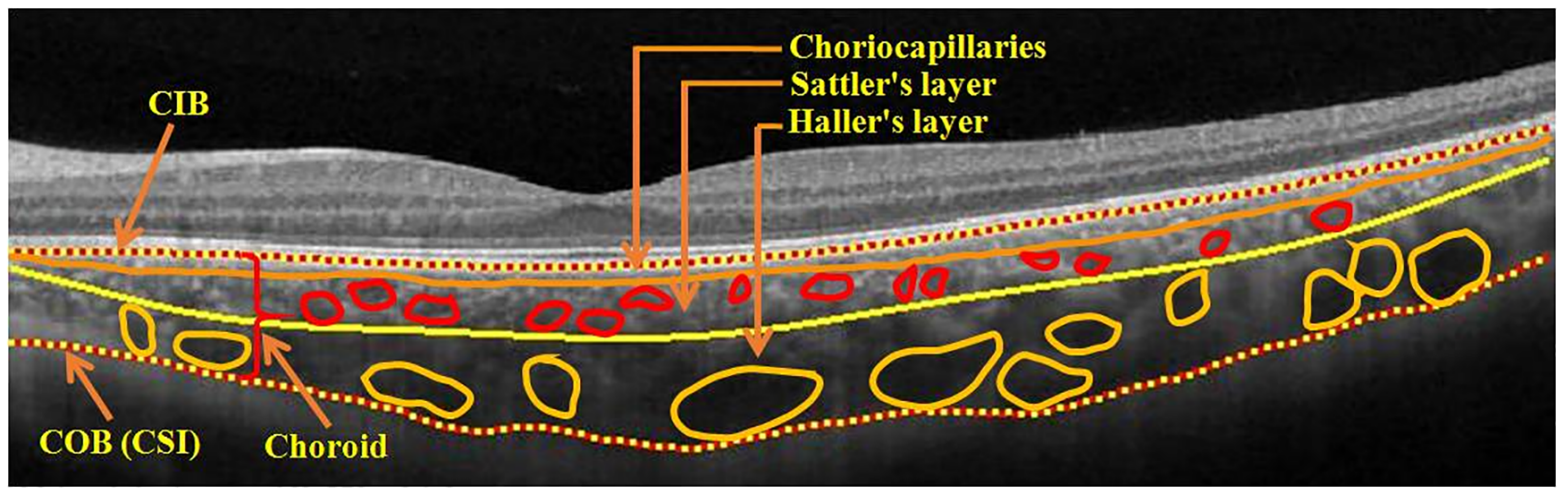
Sample swept-source OCT B-scan depicting the choroid layer and its sub-structures—Choriocapillaris, Sattler’s layer and Haller’s layer. Notation: CIB—Choroid inner boundary; COB—Choroid outer boundary; CSI—Choroid-sclera interface.

The present state-of-the-art technology of swept-source optical coherence tomography (OCT), employing a light source with increased wavelength of 1060 nm, has enhanced the quality of choroidal imaging as well as the speed of acquisition and the field-of-view [11, 12]. Such advancements have enabled clinicians to quantify specific parameters such as choroidal thickness and volume, with a view to studying their association with various diseases [2, 3, 13, 14, 15]. Yet, those studies focusing on gross structural changes do not fully exploit the available information. A better understanding of the underlying pathology can be obtained by investigating changes within choroidal sub-structures [15, 16]. Indeed, clinicians have begun to investigate the effect of various diseases on Haller’s layer [10, 17, 18]. Recently, in an assessment of choroid patterns in healthy eyes using en face OCT [19], the authors indicated that the vasculature pattern potentially plays a role in the origin and development of neuroretinal pathologies, with possible effect on chorioretinal diseases and circulatory abnormalities such as central serous chorioretinopathy (CSC) [20]. However, the above work takes a qualitative approach towards manual evaluation of choroidal morphology. Indeed, due to the intricate vasculature involved, manual quantification of Haller’s layer could be difficult, tedious, and error-prone. Against this backdrop, to obtain quantitative estimate of clinically significant parameters, it is imperative to develop a suitable algorithmic tool. Specifically, the envisioned tool should (i) segment the choroid, and (ii) delineate Haller’s layer from Sattler’s layer (see Fig 1). As algorithmic segmentation of choroid has already been achieved with high accuracy [21, 22, 23, 24, 25, 26, 27], we are left with the task of delineating between large and medium sized vessels.

So far, structural quantification of Haller’s layer has received limited attention. A method based on probability cones has recently been suggested for detecting Haller’s layer boundary in volume OCT scans [28]. However, such method makes use of volumetric heuristic, which does not apply to the more ubiquitous B-scans. To fill this gap, we propose to delineate between Haller’s and Sattler’s layers in individual B-scans using a sequence of 2D processing steps. Our 2D technique is less complex, yet extendable to volumetric quantification. Interestingly, evaluation of algorithmic performance also presents significant challenge as ground truth information generally remains unavailable. In the aforementioned work [28], the algorithmic accuracy was evaluated qualitatively, by comparing the algorithmic outcome with clinically salient features observed in 2D Indocyanine Green Angiography (ICGA) images in a subjective manner. Such evaluation suffers from two limitations. Firstly, as retinal vasculature and choriocapillaries potentially occlude many large choroidal vessels in 2D ICGA images, features observed in the latter many not provide a fair basis for comparison. Further, a quantitative, rather than a qualitative, measure of accuracy would provide a more desirable basis for clinical decision making. Accordingly, we propose to adopt a quantitative index of algorithmic accuracy. Unfortunately, a direct calculation of accuracy with reference to the ground truth remains infeasible in view of the latter’s unavailability. Thus, one must choose an indirect measure of accuracy calculated vis-à-vis new appropriate reference. An analogous situation arose in the context of algorithmic characterization of the choroid (in its entirety) [27]. In that work, observer repeatability was taken as reference, with which the mean deviation between outcomes of algorithmic and manual procedures was compared. In this paper, we similarly evaluate the performance of the proposed method to algorithmically demarcate Haller’s layer in the individual B-scans.

## Materials and methods

### Experimental datasets

This observational study was conducted at L.V. Prasad Eye Institute, Hyderabad, India, with the approval of the institutional review board and followed the tenets of the Declaration of Helsinki. Fifty healthy subjects (25 women and 25 men) were recruited, who consented to the OCT imaging of the ocular posterior segment. The mean age of the subjects was 50.7 years (standard deviation: 18.5 years). Further, the mean spherical equivalent was 0.1±0.9. OCT scans were acquired using a Triton swept-source OCT device (Topcon Corporation, Tokyo, Japan), which uses a tunable laser centered at 1050 nm to acquire 100,000 A-scans per second. Only central foveal scans were included in the study. Further, only central B-scan of one eye, randomly chosen, of each subject was considered.

### Manual segmentation

Segmentation results produced by the proposed algorithm were validated against manual markings. In particular, the Haller’s layer boundary was marked twice by single observer in two separate sessions masked from previous markings. To this end, the freehand selection tool of the publicly available ImageJ software was used [29]. These markings were used to estimate observer repeatability and to validate the robustness of the proposed algorithm.

### Proposed automated methodology

**Fig 2:**
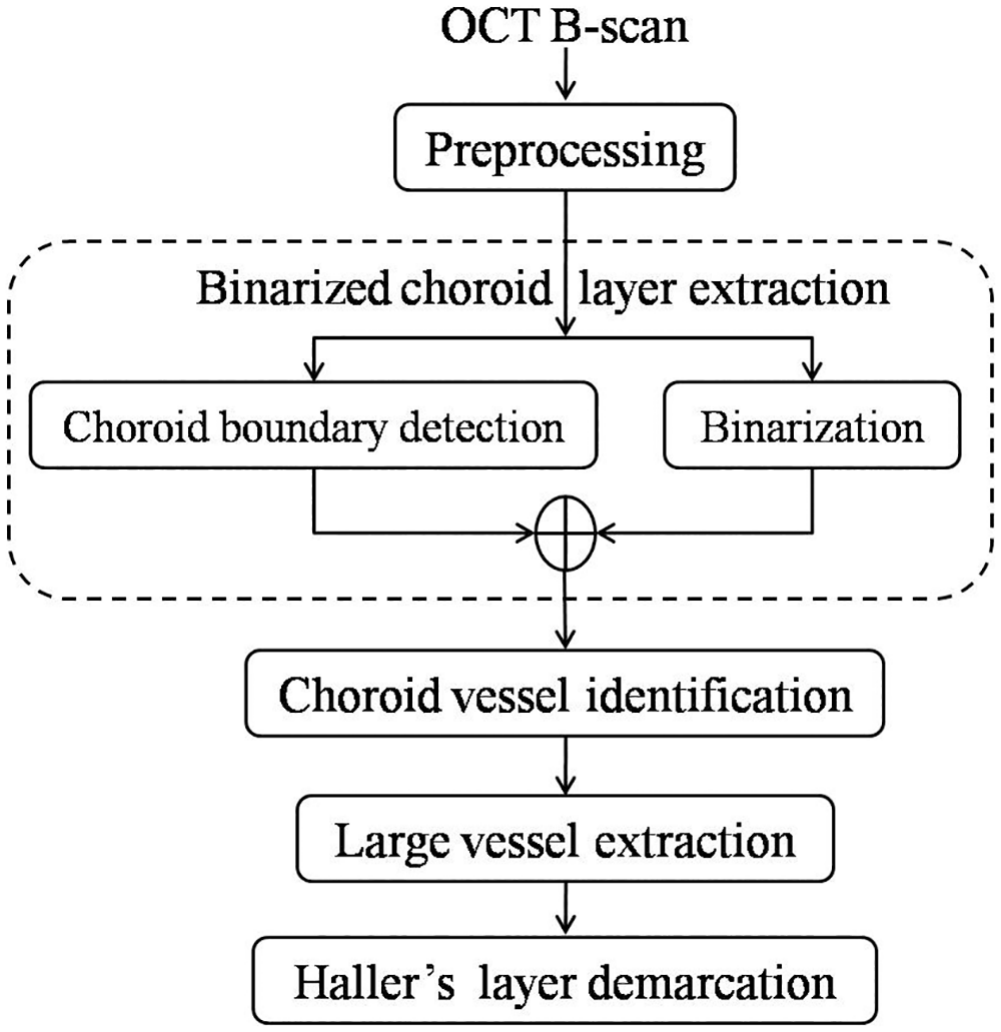
Schematic of proposed algorithm.

The proposed methodology is depicted as flow chart in Fig 2. The algorithm mainly involves extraction of binarized choroid layer, choroid vessel identification and large vessel extraction. A step-by-step description of the methodology is presented in the following.

#### Preprocessing

**Fig 3:**
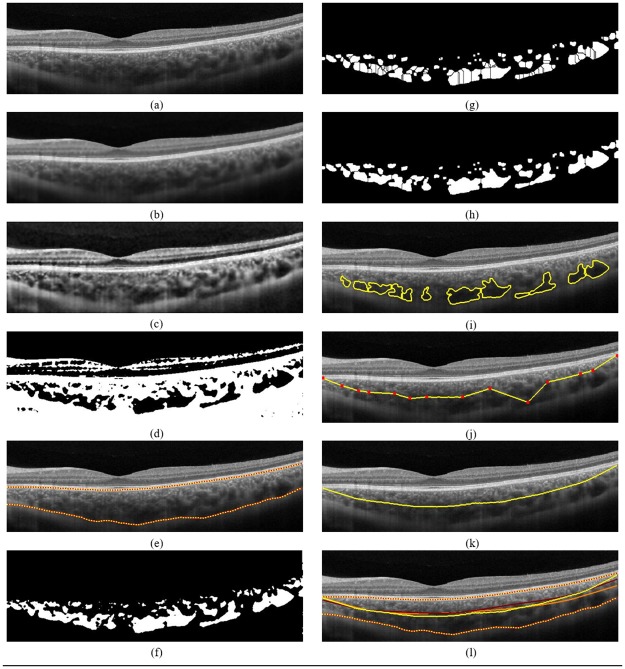
Proposed algorithm depicted graphically. (a) Raw OCT B-scan; (b) Denoised using Wiener filter; (c) Adaptive histogram equalized image; (d) Binarized image [30]; (e) Detected choroid boundaries [27]; (f) Binarized choroid layer (inverted); (g) Watershed segmentation; (h) Watershed segmentation post extended-minima; (i) Large vessel boundaries; (j) Innermost (linear interpolated) points of the vessels; (k) Smoothened Haller’s layer boundary; and (l) Algorithmic (yellow) vs (orange and red) manual demarcations.

In optical coherence tomography (OCT), the inherent speckle noise affects the visibility of various tissues and may confound the segmentation [31]. Further, due to attenuation of the signal when penetrated into deeper structure such as choroid, the contrast between vessel region and stromal regions is not high. In view of this, we first convolved the raw OCT B-scan with a 5 × 5 Wiener filter which is already shown to reduce the speckle and increase the signal-to-noise ratio, while preserving strong edges [32]. Fig 3b depicts the wiener filtered estimate of raw OCT scan shown in Fig 3a. Subsequently, adaptive histogram equalization was applied, with 8 × 8 tiling, for contrast enhancement (see Fig 3c).

#### Binarized choroid layer extraction

Next, we proceed to binarization of the OCT image to extract the choroid vessels. To this end, we adopted the recently published binarization method based on exponential enhancement [33] and additional nonlinear processing [30] (see Fig 3d). At this point, we localize choroid layer in the binarized image thus obtained by detecting the choroid inner boundary (CIB) and choroid outer boundary (COB) (refer to Fig 1) algorithmically from the preprocessed OCT image. In particular, we adopted the structural similarity (SSIM) index based methodology previously validated by our group, which reported more than 95% Dice coefficient vis-à-vis manual delineation [27]. Accordingly, the boundaries obtained for the representative image at hand is depicted in Fig 3e and the corresponding binarized choroid layer segmented from the previously obtained binarized image is shown in Fig 3f. Notice that in the binarized choroid layer (BCL), dark regions indicate the choroid vessel cross-sections and the white regions indicate the stroma.

#### Choroid vessel identification

In the BCL obtained earlier, due to tightly packed vasculature, often times multiple vessels appear connected and hence may result in spurious detections. In response, we attempted to delineate such connected vessel cross-sections. To this end, we begin with complementing the BCL, i.e., marking vessels cross-sections as white pixels, for ease of processing (see Fig 3g). Subsequently, we performed morphological closing operation, with a structuring element of size 5 × 5 pixels, to remove tiny spurious structures. Next, watershed-distance transformation is applied on the binary image to delineate the connected vessels [34]. In particular, first the distance transform (DT) is obtained which computes the distance from every pixel to the nearest nonzero-valued pixel. Generally, distance transform should be followed with watershed transform (WT) which detects the vessel boundaries. However, in this case, applying watershed immediately after DT would result in over segmentation (Fig 3h). In view of this, we performed extended-minima transform prior to WT to avoid such complexities (Fig 3i). The zero valued pixels (*w*) of WT estimate signify the watershed ridge pixels which in turn indicate the initial vessel boundaries (Fig 3i). Finally, a logical AND operation between the BCL and the *w* extracts complete the segmentation (Fig 3j) [34].

#### Large vessel extraction

We now proceed to categorize the segmented vessel cross-sections to obtain the large vessels. We acknowledge that there is no set criteria for separating the large vessels [3]. However, inspired by structural properties of choroid, we proposed a two fold approach to identify the large vessels. In the first step, all the vessels lying along the choroid-sclera interface (CSI) within 5 pixels are considered as vessels belonging to Haller’s layer [10]. In the next step, among the remaining cross-sections only those vessels that have area greater than the median of the cross-sectional areas identified along the CSI are also considered as large vessels.

#### Haller’s layer demarcation

Finally, the cross-sections obtained from the union of the aforementioned two steps (Fig 3g) are used to demarcate the Haller’s layer from Sattler’s layer. In particular, innermost pixel from each cross-section (Fig 3h) is identified and subsequently, a two-stage interpolation is performed. In the first stage, we applied a linear interpolation to obtain an initial estimate of Haller’s layer boundary (Fig 3i). In the second stage, we employed a non linear interpolation, based on robust local weighted linear least squares regression (with a window size equal to one fifth of the width of the B-scan), to obtain a smooth estimate (Fig 3j).

### Statistical measures

Accuracy of proposed methodology was evaluated by comparing algorithmic segmentation vis-à-vis manual markings performed twice by a single observer in separate sessions in a masked fashion. Specifically, we adopted certain standardized measures established earlier for evaluating algorithmic segmentation of choroid [27]. In particular, we evaluated absolute difference (AD), correlation coefficient (CC) and Dice coefficient (DC) between algorithmic segmentation and the manual reference i.e., average of two manual markings. Further, these values were compared against corresponding values obtained for intra-observer repeatability. Finally, quotient measures—quotient of mean and quotient of coefficient of variance, were also obtained to facilitate comparison against different algorithms. In particular, quotient measures close to value ‘one’ are desired which indicate that automated algorithm performs at par with manual performance. The definitions and further details of all the measures are presented in S1 Appendix provided in Supplementary material.

## Results

**Fig 4:**
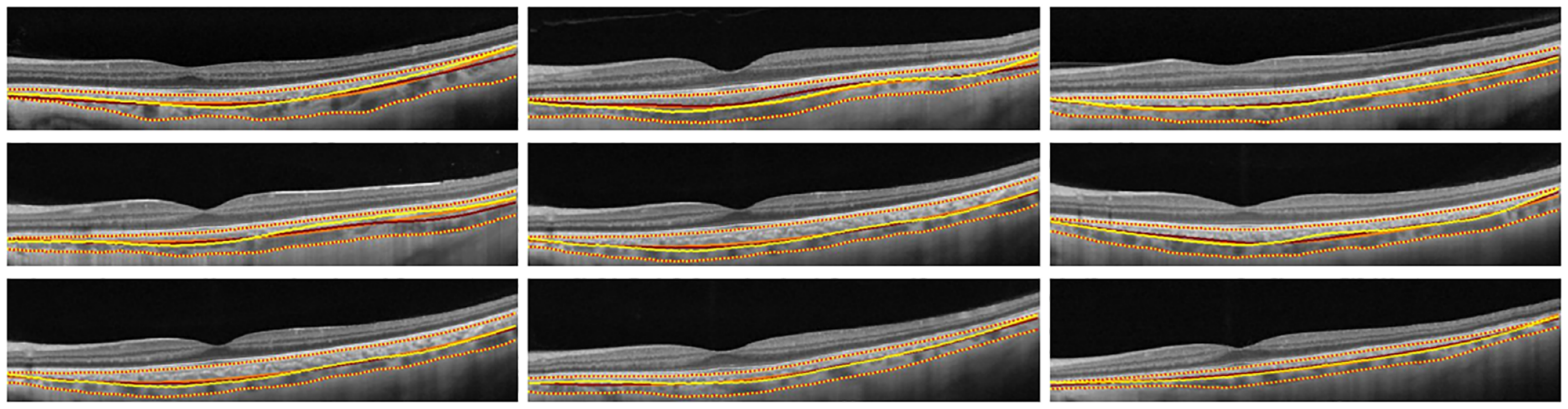
Automated vs manual markings of Haller’s layer; yellow—Automated, orange—Manual marking by grader-1, red—Manual marking by grader-2, yellow-red stripes—Choroid inner and outer boundaries.

We now proceed to evaluate the accuracy of the proposed algorithm. In particular, on 50 B-scans of 50 eyes from unique subjects, our methodology was tested. The mean age of the subjects was 50.7 years (standard deviation: 18.5 years). Further, 25 subjects were women, and 25 subjects were men. The mean spherical equivalent was 0.1 ± 0.9. Fig 4 depicts close agreement between the algorithmic segmentation and manual markings on nine representative B-scans. Next, we turn to evaluate the proposed methodology quantitatively.

### Statistical performance analysis

As alluded earlier, performance of the algorithm evaluated against the manual markings using the metrics—Absolute difference (AD), correlation coefficient (CC), Dice coefficient (DC), and performance quotients are presented here.

#### Absolute difference

**Fig 5:**
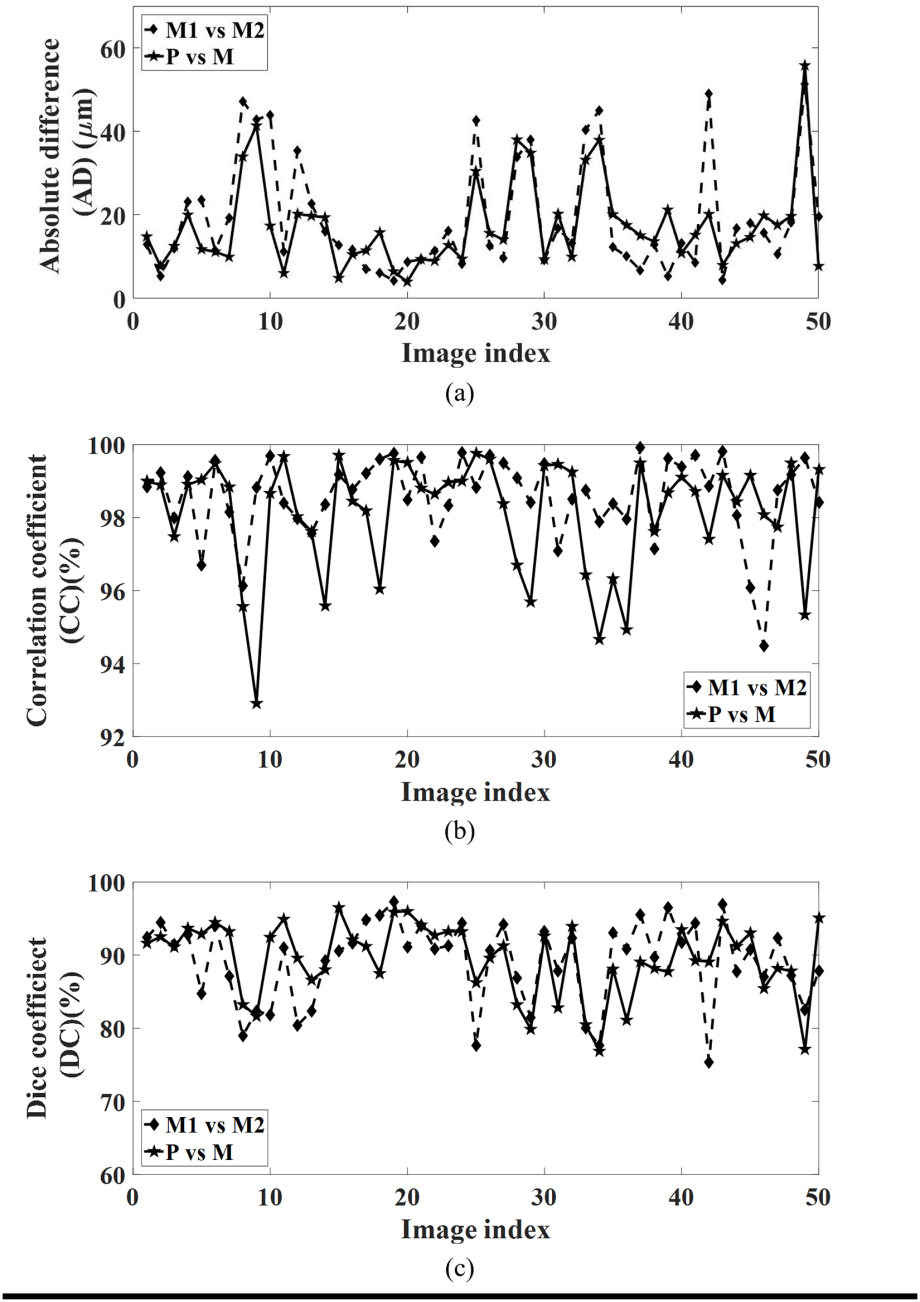
Statistical analysis: Automated vs manual methods. Notation: M1—manual segmentation-1, M2—manual segmentation-2, M—average of M1 and M2, P—proposed.

On the 50 B-scans, absolute difference estimated between algorithmic segmentation and manual reference spans from 0.0009 *μ*m to 113.75 *μ*m with a mean of 17.54 *μ*m and standard deviation (SDAD) 16.45 *μ*m. These values were observed to be in close agreement with the corresponding observer repeatability values ranging from 0.0011 *μ*m to 119.54 *μ*m with a mean 19.19 *μ*m and SDAD 17.69 *μ*m. The mean AD (MAD) and SDAD values suggest that algorithm was more consistent than the observer repeatability. Fig 5(a) depicts absolute difference estimates averaged per scan between algorithmic and manual reference alongside observer repeatability values. Finally, the quotient measures, quotient of mean (QMAD) and quotient of coefficient of variation (QCVAD) were observed to be close to 1.0 with values 0.91 and 1.02, respectively, corroborating the efficacy of the algorithm. Further details are furnished in Table 1.

**Table 1:** Statistical analysis: Automated vs manual methods. Notation: M1-manual segmentation-1, M2-manual segmentation-2, M-average of M1 and M2, P-proposed, M*q*-mean, SD*q*-standard deviation, CV*q*-coefficient of variation, QM*q*-quotient of mean, QCV*q*-quotient of coefficient of variation, *q* takes AD, CC or DC.

Evaluation criteria	Method	Parameter	Unit	Value
Absolute difference (AD)	Automated (P vs M)	MAD (SDAD)	*μ*m (*μ*m)	17.54 (16.45)
		Min—Max	*μ*m	0.0009–113.75
		CVAD	ratio	0.9379
	Manual (M1 vs M2)	MAD (SDAD)	*μm* (*μm*)	19.19 (17.69)
		Min—Max	*μ*m	0.0011–119.54
		CVAD	ratio	0.9218
		QMAD	ratio	0.91
		QCVAD	ratio	1.02
Correlation coefficient (CC)	Automated (P vs M)	MCC (SDCC)	% (%)	98.10 (1.60)
		Min—Max	%	92.92–99.76
		CVCC	ratio	0.0163
	Manual (M1 vs M2)	MCC (SDCC)	% (%)	98.59 (1.12)
		Min—Max	%	94.48–99.91
		CVCC	ratio	0.0114
		QMCC	ratio	1.35
		QCVCC	ratio	1.43
Dice coefficient (DC)	Automated (P vs M)	MDC (SDDC)	% (%)	89.48 (5.03)
		Min—Max	%	76.88–96.47
		CVDC	ratio	0.0562
	Manual (M1 vs M2)	MDC (SDDC)	% (%)	89.12 (5.68)
		Min—Max	%	75.35–97.34
		CVDC	ratio	0.0637
		QMDC	ratio	0.97
		QCVDC	ratio	0.88

#### Correlation coefficient

Turning to comparison in terms of correlation coefficient (CC), on the dataset at hand, the proposed algorithm achieved a high CC, ranging between 92.92% and 99.76% with a mean of 98.10% and a standard deviation (SDAD) 1.60%, with manual reference. On the other hand, the CC among two manual markings was ranging from 94.48% to 99.91% with a mean of 98.59% and SDAD of 1.12% demonstrating a high intra-observer repeatability as well as close match with proposed algorithm. Fig 5(b) depicts the CC comparison automated algorithm vis-à-vis manual markings. Proceeding in similar vein as earlier, we obtained the quotient of mean (QMCC) and quotient of coefficient of variation (QCVCC) which were also close to 1.0 with values 1.35 and 1.43. Comprehensive details are provided in Table 1.

#### Dice coefficient

Similar observations, mimicking AD and CC, are also made with Dice coefficient (DC) analysis. In particular, the DC values estimated between algorithmic segmentation and manual reference spanned between 76.88% to 96.47% with a mean of 89.48% and standard deviation (SDDC) 5.03% while the DC between manual demarcations was observed to vary between 75.35% to 97.34% with a mean 89.12% and SDAD 5.68%. Fig 5(c) depicts DC between algorithmic estimates and manual reference against corresponding observer repeatability values. The quotient of mean (QMDC) and the quotient of coefficient of variation (QCVDC) were found to be close to 1.0 with values 0.97 and 0.88, further substantiating the accuracy of the automated algorithm. Table 1 presents further details.

Overall, in light of the above observations, the proposed algorithm was shown to have a close agreement with manual reference and demonstrated better performance than the intra-observer repeatability.

## Discussion

In this work, we proposed an automated methodology to detect the boundary separating Haller’s and Sattler’s layers of the choroid with an aim of quantifying Haller’s layer. Specifically, we described an intuitive heuristic to identify the large blood vessels after binarizing the choroid layer. Delineation results of our algorithm exhibit close agreement with those obtained manually, specifically achieving a Dice coefficient of 89.48% vis-à-vis an intra-observer Dice coefficient of 89.12%. In fact, to the best of our knowledge, ours remains the first attempt at quantitative performance measurement comparison for the problem at hand. An earlier algorithm in similar circumstances was evaluated only via subjective quality comparison with 2D ICGA images [35]. Here, note that retinal sublayers, with boundaries marked by significant intensity gradient, are relatively straightforward to delineate [36, 37, 38]. In contrast, segmentation of choroidal substructures poses a tougher challenge. Unlike in retinal sublayers, choroidal sublayers are not defined by sharp intensity transition and potentially possess structural similarity. Consequently, traditional gradient-based segmentation techniques, employed for quantifying retinal layers, are not appropriate. The present method, employing extended-minima transform alongside watershed segmentation, has been developed to meet such a requirement.

Further, there is no ground truth or absolute criterion for categorizing the large and medium sized vessels. Classification between those must be based on relative diameter of the blood vessels rather than a predetermined threshold.

In rare instances, our method could result in intermediate introduction of deformities and warping in vessel cross-sections, potentially leading to spurious delineations. However, such demarcations are unlikely to affect the estimated boundary between large and medium-sized vessels because the proposed median criterion removes outliers and ensures robustness. However, in view of the intricate nature of the choroid vasculature, it is conceivable that successful criteria, other than the median criterion, could be developed to differentiate between large and medium-sized blood vessels. However, we expect the performance of any such algorithm irrespective of the underlying classification criterion, to still be dictated by the signal-to-noise ratio of the OCT images. For instance, in a 256 scan volume acquired without averaging from a Triton swept-source machine, the contrast between stromal and luminal regions is potentially low, and hence, may adversely affect segmentation. In such cases, further preprocessing could improve final outcome.

In the current study, the proposed algorithm was tested only on healthy eyes. Next, we plan to test our algorithm on images taken from diseased subjects, specifically, including those suffering from age-related macular degeneration (AMD) and central serous chorioretinopathy. However, complex structural changes in diseased eyes may prevent application of the proposed algorithm in its current form, and require suitable modification. We shall also extend our method to volumetric analysis of large and medium-sized choroidal vessels. A preliminary work tracing choroidal vessels in 3D has recently been reported [39].

## Supporting information

S1 Appendix Performance criteria and statistical measures. (PDF)
