# Supplementary material for: Automated quantification of Haller’s layer in choroid using swept-source optical coherence tomography
Source: PLoS One. 2018 Mar 7;13(3):e0193324. doi: 10.1371/journal.pone.0193324 (PMC5841756; doi:10.1371/journal.pone.0193324)
Supplement: S1 Appendix — (PDF) [file pone.0193324.s001.pdf]

S1 Appendix. Performance criteria and statistical measures

A Performance criteria

Metrics including absolute difference (AD), correlation coefficient (CC), and Dice coefficient (DC) are established as standard for comparing two segmentations obtained by different methods, in a similar problem[1] and are reproduced in the following.

1. Absolute difference: Suppose x_i and y_i denote the thickness values at the i-th $(i = 1, ..., N)$ column (A-scan index) in two measurements. Then the absolute difference (AD) between those measurements at the i-th column are respectively given by

$$\mathrm{AD}_i = |x_i - y_i|. \tag{A.1}$$

2. Correlation coefficient: For two measurements x_i and y_i, $i = 1, ..., N$, seen earlier, the correlation coefficient (CC) is defined by

$$CC = \frac{\sum_{i=1}^{N} x_i y_i}{\sqrt{\sum_{i=1}^{N} x_i^2 \sum_{i=1}^{N} y_i^2}}. \tag{A.2}$$

3. Dice coefficient: Denote the respective sets of pixel indices in the i-th $(i = 1, ..., N)$ column of two segmentations by P_i^x $(|P_i^x| = x_i)$ and P_i^y $(|P_i^y| = y_i)$. Then the Dice coefficient (DC) is defined by

$$\mathrm{DC} = \frac{2\sum_{i=1}^{N} |P_i^x \cap P_i^y|}{\sum_{i=1}^{N} |P_i^x| + \sum_{i=1}^{N} |P_i^y|}. \tag{A.3}$$

B Statistical performance measures

For a collection $\{q_i\}_{i=1}^{N}$ of samples of quantity q (either AD, CC or DC), its mean Mq, standard deviation (SD) SDq, and coefficient of variation (CV) CVq are defined by

$$\mathrm{M}q = \frac{1}{N}\sum_{i=1}^{N} q_i, \qquad \mathrm{SD}q = \sqrt{\frac{1}{N}\sum_{i=1}^{N}(q_i - \mathrm{M}q)^2}, \qquad \mathrm{CV}q = \frac{\mathrm{SD}q}{\mathrm{M}q}, \tag{B.1}$$

[1] Various metrics were standardized for evaluating algorithmic accuracy for choroid segmentation in the publication: Vupparaboina KK, Nizampatnam S, Chhablani J, Richhariya A, Jana S. Automated estimation of choroidal thickness distribution and volume based on OCT images of posterior visual section. Com- puterized Medical Imaging and Graphics. 2015;46:315–327.

which respectively measure the central tendency, the dispersion, and the standardized dispersion.

C Performance quotients

Quotient measures, quotient of mean and quotient of coefficient of variance, are proposed to evaluate algorithmic performance in relation to observer repeatability and facilitate comparison with reported algorithms.

1. Quotient of mean (QM): The quotient of mean, QMq, is defined by the ratio

$$\mathrm{QM}q = \frac{|\mathrm{M}q^{auto} - q^{ideal}|}{|\mathrm{M}q^{ref} - q^{ideal}|}, \tag{C.1}$$

where Mq^{auto} and Mq^{ref}, respectively, indicate the mean values obtained by the automated and manual delineations, while q^{ideal} denotes the ideal value of q. A low QMq value is desirable. Specifically, QM$q = 1$ would make the accuracy of the automated algorithm indistinguishable from observer grading in terms of mean error.

2. Quotient of coefficient of variance (QCV): Quotient of coefficient of variance, QCVq, is defined by

$$\mathrm{QCV}q = \frac{\mathrm{CV}q^{auto}}{\mathrm{CV}q^{ref}}, \tag{C.2}$$

where CVq^{auto} and CVq^{ref}, respectively, indicate the CV obtained by the algorithm and that obtained manually. Again, a value close to one is desired for QCVq and the value QCV$q = 1$ would make the algorithm indistinguishable from the manual methods. As alluded earlier, the general quantity q in (C.1) and (C.2), can specifically be either AD, or CC, or DC.
